# polyGR produced by CASP8 GGGAGA repeat expansion increases phosphorylation of Tau

**DOI:** 10.1002/alz70855_102925

**Published:** 2025-12-23

**Authors:** Rodrigo Francisco Tomas, Huong T. Phuong, Logan R. Bell, Cemal Akamese, Lien Nguyen

**Affiliations:** ^1^ University of Florida, College of Medicine, Gainesville, FL, USA; ^2^ University of Florida, Gainesville, FL, USA

## Abstract

**Background:**

Alzheimer's disease (AD) affects 6.5 million people in the United States with cases doubling by 2050. The accumulation of intracellular phosphorylated Tau tangles and amyloid beta plaques are pathological hallmarks of AD, however, the cause of these pathologies in most sporadic AD cases (∼95%) remains unclear. Familial studies propose that genetic factors are a main contributor, but further investigation is needed to understand the cause of AD. Our previous work showed polyGR‐positive aggregates frequently accumulate in sporadic AD autopsy brains. We identified a repeat expansion (RE) mutation within a SINE/VNTR/Alu (SVA) element located in the *CASP8* gene that produces polyGR‐containing proteins. A rare sequence variant of the *CASP8* RE, *CASP8*‐GGGAGA‐AD‐R1, is associated with increase in AD risk (odds ratio of 2.2; *p* = 3.1x10^‐5^). Interestingly, overexpression of *CASP8*‐GGGAGA‐AD‐R1 led to increases in aggregation of the polyGR‐containing protein and cell toxicity. Here we aim to dissect disease mechanisms involved in *CASP8*‐GGGAGA‐AD‐R1's increased toxicity and possible contributions of *CASP8* polyGR‐containing proteins in AD.

**Method:**

SH‐SY5Y cells were transfected with constructs that contain the RE variant in *CASP8*‐GGGAGA‐AD‐R1 or *CASP8*‐GGGAGA common variants to express *CASP8* polyGR‐containing proteins. Our preliminary data showed that increased polyGR protein levels are detected in AD cases that experienced brain injuries or high blood pressure. To study the effects of stress, transfected cells were treated with hydrogen peroxide (H2O2) to understand the role of cellular stress on polyGR expression, which was previously shown to increase RAN translation. Changes in polyGR‐containing protein, phosphorylated tau, and caspase protein levels were studied using double immunofluorescence.

**Result:**

We detected increases in caspase‐8 (1.9‐fold, *p* = 0.0004) and *p*‐tau (2.4‐fold, *p* <0.0001) in polyGR positive cells when compared to negative polyGR cells. Interestingly, caspase‐3 protein levels do not show a similar increase. Transfected cells treated with H2O2 that induce oxidative stress result in an increase in *CASP8* polyGR protein levels (16‐fold, *p* <0.0001) and pTau signal (1.5‐fold, *p* = 0.0445).

**Conclusion:**

In summary, our results demonstrate polyGR aggregates can contribute to tau pathology and activation of caspase‐8 related pathways in AD. Additionally, increased oxidative stress favors the accumulation of polyGR‐containing proteins and related pathways, leading to increased AD pathological hallmarks.

